# How Ocean Acidification Makes Marine Fish Reproduction Vulnerable: Physiological Mechanisms and Ecosystem Consequences

**DOI:** 10.3390/toxics14090834

**Published:** 2026-09-20

**Authors:** Zihao Chen, Ruiheng Qu, Cheng Zhao, Monia Perugini, Waliullah Masroor, Quanquan Cao

**Affiliations:** 1 College of Animal Science and Technology, Sichuan Agricultural University, Chengdu 611130, China; 2 Animal Husbandry and Veterinary College, Jiangsu Vocational College of Agriculture and Forestry, Jurong 212400, China; 3 Faculty of Bioscience and Agro-Food and Environmental Technology, University of Teramo, 64100 Teramo, Italy; mperugini@unite.it; 4 Faculty of Marine Sciences, Lasbela University of Agriculture, Water & Marine Sciences (LUAWMS), Uthal 10250, Pakistan

**Keywords:** ocean acidification, pH, gonad, fish, reproductive system, HPG axis, gametogenesis, climate change

## Abstract

Ocean acidification, driven by the absorption of anthropogenic carbon dioxide into seawater, poses a significant threat to marine environments and biodiversity. This comprehensive review examines the specific impacts of acidification on the reproductive physiology of fish, integrating recent advances in our understanding of gonadal development, gametogenesis, and early embryonic development. We synthesize current findings on how altered carbonate chemistry disturbs the reproductive system from gametogenesis and fertilization to larval development and beyond. Our analysis reveals that ocean acidification affects multiple physiological pathways, including disruption of the hypothalamic–pituitary–gonadal (HPG) axis, impairment of calcium signaling, and alterations in sex hormone synthesis. We propose a mechanistic model in which pH reduction may weaken *Dax1*-mediated repression of P450arom transcription, thereby dysregulating aromatase activity and leading to sex hormone imbalance that impairs gonadal development; however, this proposed mechanism requires direct experimental validation under ocean acidification, as the net outcome may be modulated by additional regulatory layers such as epigenetic modifications and other transcription factors. Furthermore, we discuss the synergistic effects of ocean acidification with temperature elevation and other stressors, which often exacerbate reproductive dysfunction. This review concludes that no multi-generational study has yet evaluated HPG axis disruption to population-level recruitment failure, a critical gap for fishery resource assessment. We emphasize the critical importance of multi-generational and field-based studies to fully understand these impacts, which is essential for forecasting consequences for global marine biodiversity and the long-term sustainability of fisheries.

## 1. Introduction 

Since the Industrial Revolution, human activities have substantially increased atmospheric carbon dioxide (CO_2_) concentrations, rising from approximately 280 ppm in pre-industrial times to over 420 ppm in 2024 [1]. The oceans absorb approximately one-third of this anthropogenic CO_2_ through air–sea exchange, leading to ocean acidification. This process has caused a decrease in seawater pH of about 0.1 units since pre-industrial times [2,3]. This pH decline corresponds to a 25–30% increase in hydrogen ion concentration and a disruption of the seawater carbonate buffer system, which regulates the availability of carbonate ions (CO_3_^2−^). This negatively affects the calcification of marine organisms [3,4,5].

Under the Intergovernmental Panel on Climate Change (IPCC) high-emission scenario (RCP 8.5), the ocean’s pH is projected to decline by an additional 0.4 units by 2100, representing a total decline of 0.5 pH units from pre-industrial conditions [6,7]. This scenario also predicts a 0.67 m rise in global sea level and a 2.73 °C increase in sea surface temperature, respectively. The oxygen content may drop to 3.48% [8]. These physicochemical changes will inevitably affect organismal physiology, phenology, and ultimately, community structure and food webs.

The biological consequences of ocean acidification are profound, affecting marine organisms across all levels of biological organization [5,9]. Marine calcifier organisms that depend on CaCO_3_ for their shells and skeletons are especially vulnerable to reduced pH and altered carbonate chemistry [3,4]. This vulnerability arises because CO_2_ dissolution forms carbonic acid, which lowers pH and depletes the carbonate ions (CO_3_^2−^) essential for calcification [3]. Consequently, ocean acidification can increase the failure rate of calcification and accelerate the dissolution of calcium carbonate structures in corals and many shell-forming species. Conversely, certain resilient or invasive species may adapt or expand their ranges, ultimately shifting species distributions, abundances, and ecosystem structure [4,10].

For fishes, the potential consequences of ocean acidification include reduced fertility, altered reproductive behaviors, and diminished offspring survival, which may contribute to population declines and shifts in species distributions [11,12,13,14,15]. Acidification can also impair developmental processes such as bone and otolith formation in juvenile fishes, thereby reducing their ability to maintain orientation and locate prey [12,16,17]. Furthermore, because planktonic communities are key food sources for many fish larvae, the modification in plankton abundance and composition can affect larval and juvenile survival, growth, and adult reproductive performance. Together, these effects can alter fish community structure, especially in vulnerable ecosystems such as coral reefs [18,19].

Fish reproduction, i.e., gonad development, gametogenesis, spawning, fertilization, and embryonic development, involves complex, interconnected processes that are highly sensitive to environmental change [15,20]. A growing body of evidence indicates that ocean acidification can disrupt these processes, thereby threatening reproductive success, fishery productivity, and the stability of marine ecosystems [21,22,23]. Sexual reproduction, a costly process vital for species survival and evolution, is tightly regulated by environmental cues that trigger maturation and breeding [15,20]. Because these same conditions are crucial for offspring survival, rapid environmental changes can directly alter reproductive timing and success, potentially jeopardizing the long-term sustainability of diverse fish populations.

**Figure 1:**
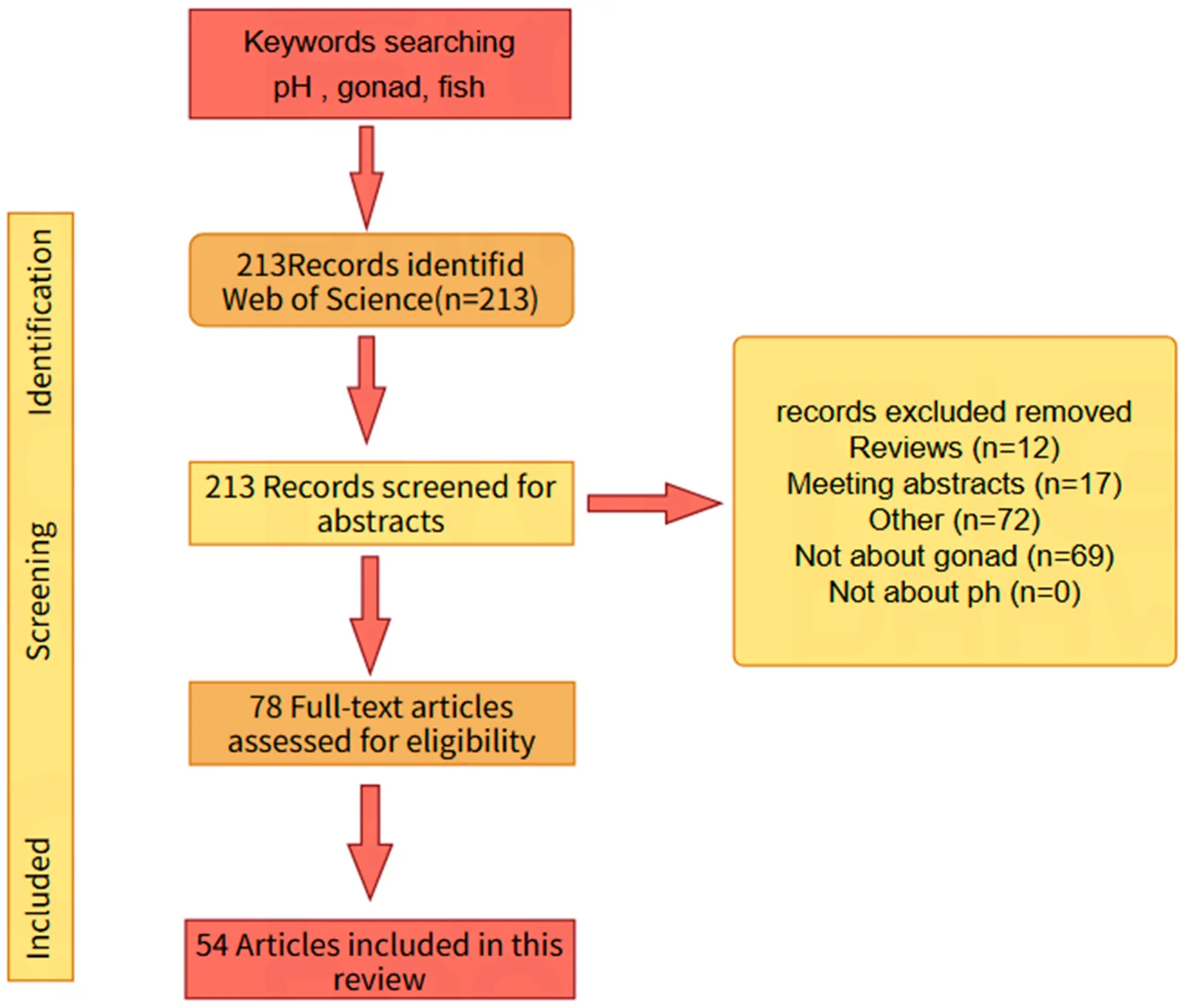
Flowchart of literature search and screening process. Values indicate the number of records identified and retained at each stage. Studies were considered relevant if they specifically investigated pH effects on fish reproductive development. Exclusion criteria included: monographs, review articles, non-fish studies, and conference abstracts. Studies lacking quantitative data (e.g., sample size, variance measures, or specific experimental results) were classified as ‘unqualified’ and excluded.

This review synthesizes the current understanding of ocean acidification effects on fish reproductive physiology, population dynamics, and ecosystem health. We examine mechanisms from molecular to organismal levels, consequences for gamete quality and embryonic development, and broader ecological implications, while identifying knowledge gaps and future research priorities for marine biodiversity conservation and management. To assess the empirical foundation of these effects, a systematic literature search was conducted across Web of Science, Google Scholar, and PubMed using keywords, including ‘ocean acidification,’ ‘reproductive system,’ and ‘gonad’ (Figure 1). Of more than 150 peer-reviewed publications initially identified, 54 met our inclusion criteria (quantitative data, peer-reviewed, fish-focused) and were synthesized here.

## 2. The Impact of Ocean Acidification on Gametogenesis

### 2.1. Gonadal Development and Maturation

The development and maturation of gonads are critical stages in the reproductive cycle of marine fish, directly determining population recruitment and sustainability [15]. These processes are increasingly threatened by ocean acidification (OA), which imposes significant metabolic and physiological stress on aquatic organisms [24,25]. Emerging research indicates that OA-induced hypercapnia triggers a shift in energy allocation; fish must divert metabolic resources toward acid–base regulation and ion homeostasis to mitigate acidosis, often at the expense of reproductive investment [24,25,26]. This ‘energy trade-off hypothesis’ posits that the elevated energetic costs of maintaining internal acid–base balance constrain the energy available for gonadal development and gametogenesis.

Elevated pCO_2_ has been shown to disrupt the hypothalamic–pituitary–gonadal (HPG) axis, leading to reduced levels of sex steroid hormones (such as 17β-estradiol) and suppressed vitellogenesis, which ultimately delays oocyte maturation [21,27]. In female fish, vitellogenesis, the accumulation of yolk in developing oocytes, is an energy-intensive process that provides essential nutrients for embryonic development. When energy is redirected toward acid–base compensation [25], vitellogenesis is suppressed, yielding oocytes of reduced size and nutritional quality [22]. These impacts are particularly acute during juvenile stages, when high metabolic demands for growth limit the capacity to compensate for pH fluctuations, potentially leading to irreversible impairment of gonadal development.

Juvenile fish are especially vulnerable to pH fluctuations because immature physiological systems and high growth-related metabolic demands limit compensatory capacity, potentially causing irreversible impairment of gonadal development [28]. Similarly, long-term warming and acidification reduced gonadal development and delayed sexual maturation in three-spined sticklebacks (*Gasterosteus aculeatus*) [22], highlighting the greater reproductive risks of chronic acidification.

### 2.2. Neuroendocrine Disruption Mechanism

Hormonal regulation is essential for fish reproduction, and ocean acidification (OA) can disrupt this process through various physiological and molecular pathways operating at multiple levels of the brain–pituitary–gonad axis [27]. OA may indirectly affect endocrine function by inducing environmental stress and redistributing metabolic energy [25]. For instance, studies demonstrate that acidified seawater can suppress insulin-like growth factor 1 (*IGF-1*) mRNA expression in juvenile zebrafish, impairing growth rates [29]. *IGF-1* is a key effector of growth hormone and plays a crucial role in promoting reproductive function at all levels of the HPG axis, including stimulation of GnRH release, increased pituitary sensitivity to gonadotropins, and direct promotion of gonadal gametogenesis and steroidogenesis [30].

OA may also alter the gene expression of the corticotropin-releasing hormone (CRH) system. While increased CRH mRNA levels are accompanied by elevated expression of its receptor (*crhr1*) and binding protein (*crhbp*), CRH secretion itself appears to be inhibited [29,31,32]. This paradoxical pattern suggests complex regulatory mechanisms at the post-transcriptional and translational levels, potentially involving alterations in neuronal depolarization or changes in neurotransmitter function [27].

The interaction between the IGF and CRH systems establishes a critical physiological checkpoint that integrates metabolic and stress signals to modulate reproduction. Under stress, corticotropin-releasing hormone (CRH), the central driver of the neuroendocrine stress response, exerts strong inhibitory effects on the HPG axis. As shown in Table 1. Specifically, CRH suppresses GnRH pulsatility, which is critical pattern of hormone secretion necessary for reproductive function, while dampening pituitary responsiveness to GnRH, and directly compromising gonadal function. Notably, these systems are mutually antagonistic: chronic stress and elevated CRH can inhibit *IGF-1* signaling, creating a dual impairment that redirects energy resources from reproduction toward survival, ultimately reducing fertility and reproductive success [33]. This stress-induced suppression of reproduction has been demonstrated in coral reef fishes exposed to near-future CO_2_ levels, where elevated CRH expression correlated with reduced reproductive output [21].

Additionally, OA can directly disrupt the hypothalamic–pituitary–gonadal (HPG) axis—the central regulator of reproduction. This axis functions through hypothalamic gonadotropin-releasing hormone (GnRH), which stimulates the pituitary release of gonadotropins (FSH and LH), driving gonadal development and gamete maturation via steroid hormone synthesis [34]. This model proposes that elevated $p{CO}_{2}$ from OA impairs ${GABA}_{A}$ receptor function by disrupting intracellular chloride homeostasis, which ultimately suppresses the synthesis and release of GnRH [13,35,36].

Such suppression at the hypothalamic level disrupts the entire downstream cascade of gonadotropin secretion, thereby compromising reproductive output [36]. This mechanism has been validated in multiple fish species, including damselfish and sea bream. While the GABA_A_ receptor hypothesis is a widely cited mechanism for OA-induced behavioral impairment, the behavioral foundation of this hypothesis has been challenged. A prominent replication study found no effect of elevated CO_2_ on the behaviors used to support this mechanism [37], leading to a formal scientific debate about methodology and reproducibility [13,38]. This controversy highlights the need for more direct and robust physiological measurements to validate the GABA_A_ mechanism, moving beyond behavioral assays.

Another proposed mechanism, though speculative, involves disruption of circadian rhythm regulation. CO_2_-induced acid–base disturbances may alter the fish circadian system and melatonin secretion patterns [27,35,39,40]; disrupted melatonin rhythms could subsequently affect GnRH expression and its regulation of gonadotropins [35,39]. Given that melatonin, synthesized in the pineal gland, coordinates seasonal breeding through its interaction with the reproductive axis [39,40], such disruption might desynchronize reproductive activity and impair optimal spawning timing. However, this pathway currently lacks direct experimental support under ocean acidification and requires validation under combined effects with other abiotic stressors [27,40]; we therefore present it as a testable hypothesis rather than a confirmed mechanism.

OA can affect the energy distribution and ecological pressure of marine ecosystems. At the same time, acidified seawater inhibits the expression of insulin-like growth factor 1 mRNA and CRH system gene mRNA, thereby affecting the promoting effect of CRH system genes on CRHR1 and CRHBP expression, as well as the inhibitory effect of CRHR1 and CRHBP on CRH system gene expression. Meanwhile, changes in GABA_A_ receptor function at high pCO2 levels can affect the release of GNRH and gonadotropins (Adapted from [28,41,42]). 

**Figure 2:**
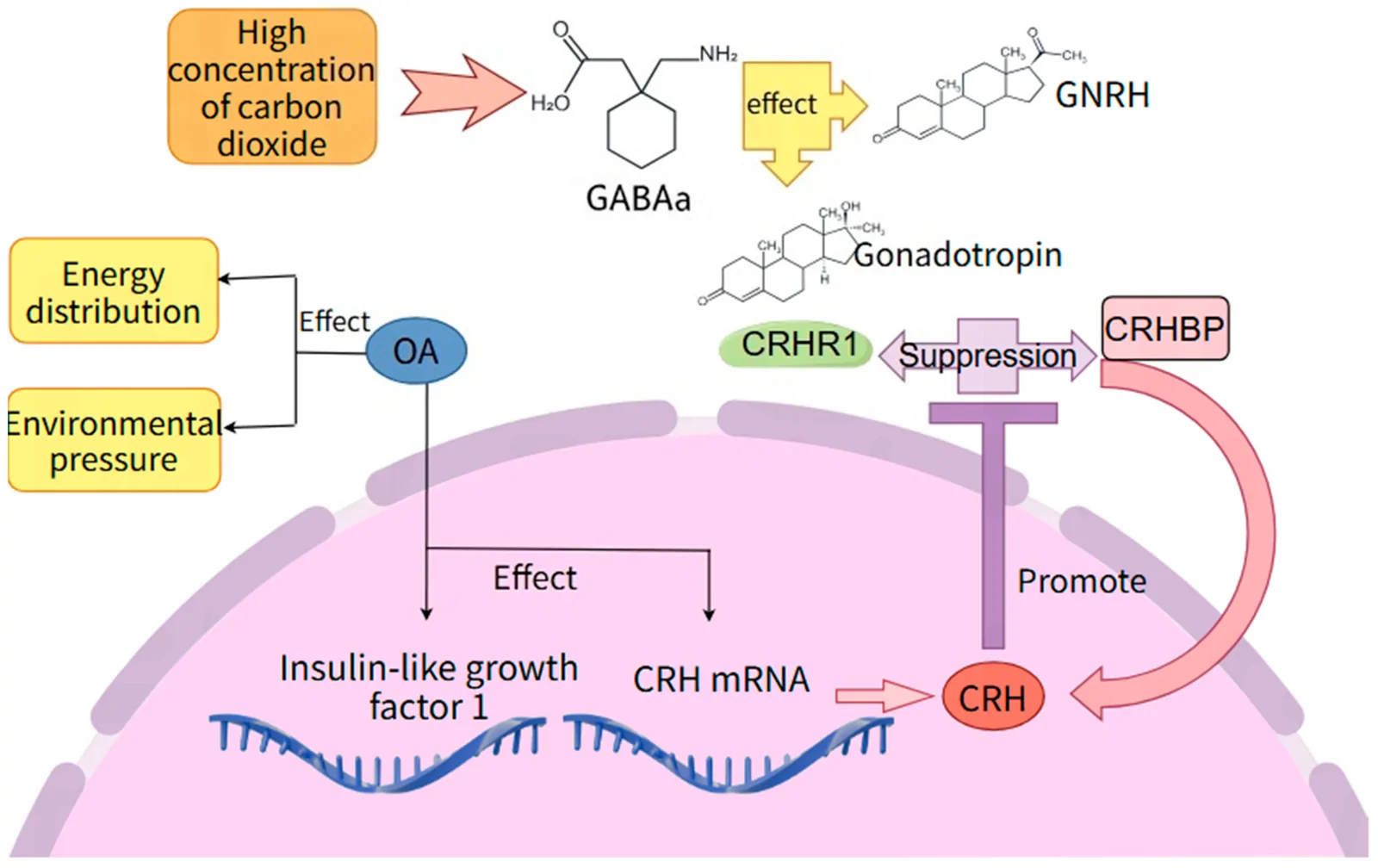
Conceptual diagram of ocean acidification (OA) impacts on fish neuroendocrine pathways. Elevated $p{CO}_{2}$ disrupts energy allocation and alters key reproductive axes. The schematic highlights the suppression of *igf-1* and *crh* system gene expression, the consequent deregulation of downstream receptors (crhr1, crhbp), and the ${GABA}_{A}$ receptor-mediated inhibition of GnRH and gonadotropin release (adapted from [28,41,42]). The illustration was created by the author using Figdraw (www.figdraw.com).

Fish reproduction is regulated by interconnected neuroendocrine pathways. Unlike tetrapods, most teleosts lack a hypothalamo–hypophysial portal system, with neurohormones instead directly innervating the pituitary [43]. Key regulators include stimulatory GnRH, inhibitory dopamine, and modulatory neuropeptides such as NPY, GABA, kisspeptin, and gonadotropin-inhibitory hormone (GnIH) [34]. These neuroendocrine signals regulate the synthesis and release of FSH and LH, with the help of gametogenesis and steroidogenesis [34,44,45,46]. A schematic summarizing of the potential mechanisms by which OA affects the CRH system and related neuroendocrine pathways is presented in Figure 2.

Cytochrome P450 aromatase (encoded by the gene *cyp19a1a*) plays a crucial role in sex hormone synthesis, particularly in estrogen synthesis. The enzyme catalyzes the conversion of androgens (testosterone and androstenedione) to estrogens (estradiol and estrone). This conversion reaction is essential for female sex determination in many fish species and for reproductive function in both sexes [47,48]. The decrease in pH caused by ocean acidification may affect the inhibitory control of P450arom expression by *Dax1* gene in ovarian follicles, thereby affecting the activity of aromatase and finally affecting the development of gonads [28,41,42].

**Figure 3:**
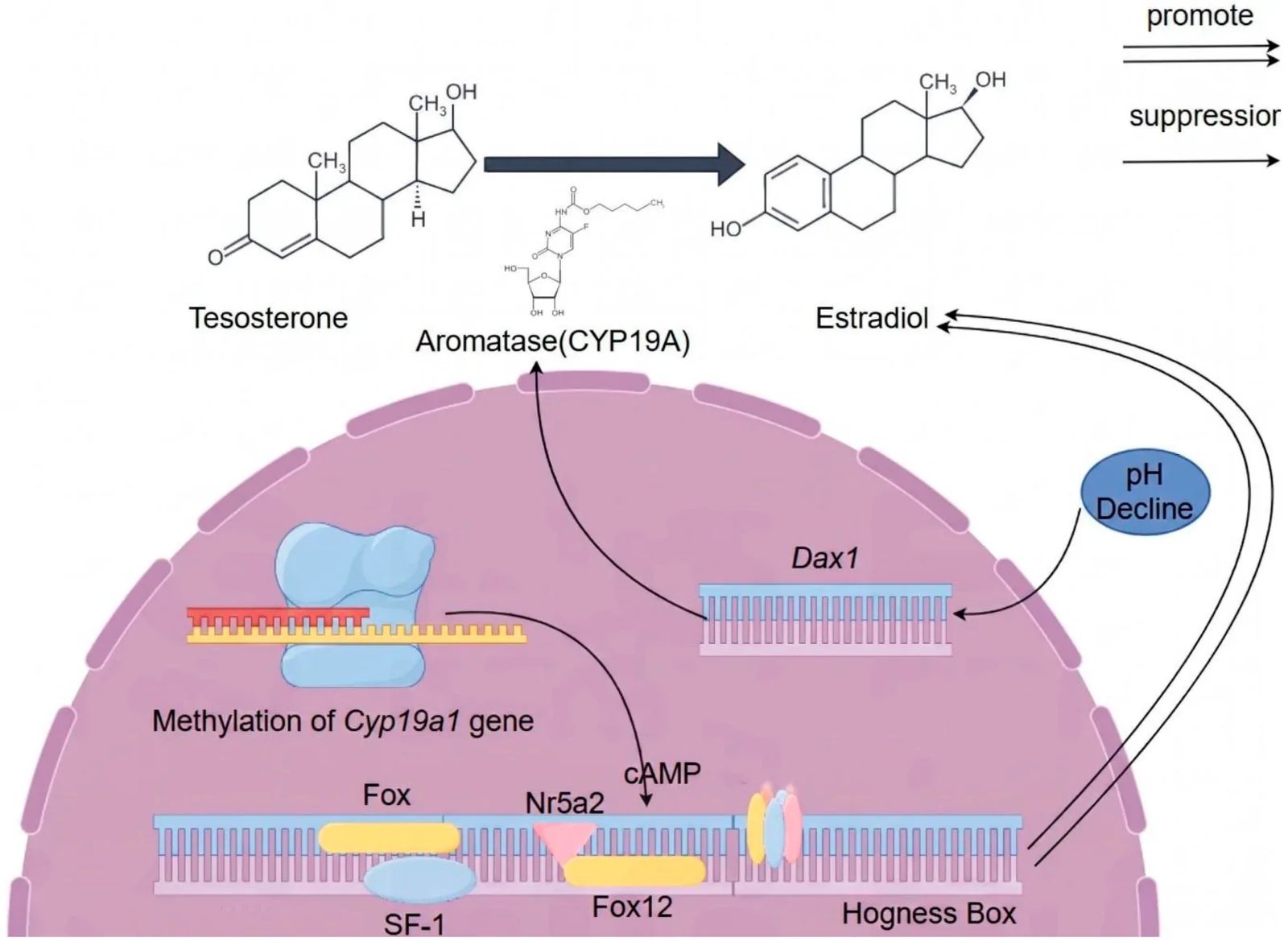
Mechanisms of pH-driven disruption on gonadal development. Decreased pH alters the regulatory pathways of *dax1* and aromatase (*cyp19a1a*) expression, impairing the irreversible conversion of androgens to estrogens and ultimately compromising fish gonadal development [41,42,49]. The illustration was created by the author using Figdraw (www.figdraw.com).

A schematic diagram summarizing the mechanisms through which decreased pH impairs gonadal development is presented in Figure 3.

*Dax1* (Dosage-sensitive sex reversal, Adrenal hypoplasia congenita, X-linked gene) encodes an orphan nuclear receptor that functions as a transcriptional repressor of P450arom expression [41,42]. OA-induced pH decline may reduce *Dax1* binding affinity or expression, weakening this repression and dysregulating aromatase activity. The resulting imbalance in sex hormone ratios can compromise gonadal development.

Environmental factors may also regulate reproductive development through epigenetic pathways. For example, in specific bony fish models, the expression pattern and methylation status of the cyp19a1 gene in the gonads have been shown to be closely related to sex determination [49]. DNA methylation is also linked to this process. These findings suggest that OA-induced pH changes could alter early sex differentiation and gonadal development. However, this mechanism requires experimental validation. Beyond direct reproductive effects, OA also impairs fish behavior and sensory function, critical for survival and reproduction, with the underlying mechanisms first elucidated [25,35].

### 2.3. The Impact on Reproductive Output

OA significantly impairs the quality and function of fish gametes, which can reduce fertilization success and offspring survival [21,22]. Fish reproductive cells are highly sensitive to environmental pH. For instance, sperm motility is optimal under slightly alkaline conditions, with a suitable range of pH 8.0–10.0 for most freshwater fish species [50]. Similarly, the pH of the ovarian fluid surrounding eggs is critical for embryonic development. In lake trout (*Salmo trutta lacustris*), the pH range yielding the highest egg survival rate (≥80%) is 8.44–8.57 [51]. Maintaining suitable pH also extends gamete functional lifespan. In rainbow trout (*Oncorhynchus mykiss*), sperm remain motile longer when ovarian fluid pH exceeds 7.4 [52], suggesting that even moderate pH declines could shorten the window for successful fertilization in broadcast spawners. OA may impair reproduction through multiple pathways, including direct gamete damage, sensory-mediated behavioral disruption, and altered Ca^2+^ signaling required for egg activation [9,53,54]. In damselfish, increased aggression and disrupted courtship under OA may further reduce mating success and recruitment [21,55] as shown in Table 2.

## 3. The Impact of OA on Fertilization and Embryonic Development

### 3.1. Impacts on Sperm Motility and Viability

Sperm motility is a key factor for successful fertilization and is highly sensitive to the pH value of the activating environment [58]. In most bony fish, sperm are inactive in the testes and seminal plasma, and their motility potential can only be activated after expulsion and dilution with external water or activating fluid, which induces changes in osmotic pressure and ionic composition [58,59]. This dependence on extracellular conditions makes sperm potentially susceptible to environmental pH shifts. However, it is important to note that the majority of studies cited below employed artificial activation media with broad pH ranges, often in freshwater species, and do not directly replicate the conditions of ocean acidification. Moreover, sperm activation in marine teleosts is governed by a complex interplay of osmolality, salinity, ionic composition, pH, and bicarbonate; thus, pH should not be considered an isolated causal variable.

Research on the Asian catfish (Clarias macrocephalus) established a direct link between optimal conditions and high fertilization rates (89–94%), achieved using an artificial seminal plasma at pH 7.8 [60]. More recent studies [61] on Brycon orbignyanus and Prochilodus vimboides show that optimizing the composition of activation solutions (e.g., pH and osmolality) is crucial for maximizing fertilization and hatching success for both fresh and frozen–thawed sperm. Similar alkaline pH optima are reported in other cyprinids (bigeye carp, color carp, red carp, and longfin basket fish), indicating that the optimal activation pH for sperm often reflects the natural pH of seminal plasma [58]. Therefore, maintaining a weakly alkaline water environment during artificial insemination can effectively improve fertilization rates in these species.

Different fish species show varying tolerance ranges when exposed to different pH levels [56,58]. Further studies have revealed that salmonids such as river trout and brown trout maintain a certain level of vitality (31–38%) at pH 5.5, while rainbow trout also exhibit tolerance to acidic environments, suggesting that some species have a broad pH tolerance range [52,56]. Species-specific pH tolerance does not eliminate potential risks, as pH and salinity also influence sperm motility in marine teleosts, including European sea bass (Dicentrarchus labrax), gilthead sea bream (Sparus aurata), and Atlantic sole [47,48,58,62,63]. Collectively, these findings indicate that pH is a significant factor affecting sperm performance, but they also underscore the need for direct experimental validation under projected ocean acidification conditions, taking into account the multifactorial nature of sperm activation and the specific characteristics of marine fish. Therefore, the vulnerability of marine fish sperm to OA-driven pH declines, discussed further below, remains a hypothesis that requires dedicated testing with environmentally realistic scenarios.

### 3.2. Changes in Egg Quality and Morphology

Low-pH environments can severely impact the early developmental stages of fish. While the studies cited here were conducted at pH levels substantially lower than those projected for ocean acidification (OA) and thus do not simulate marine OA conditions, they offer useful mechanistic insights into how extreme acidification may affect chorion integrity and embryonic development. Numerous investigations have reported that acidic conditions significantly reduce hatching rates and delay embryonic development [57,64]. For instance, in Atlantic salmon (Salmo salar), water pH of 4.5–5.0 prolonged hatching time by up to 6 days, and further reduction to pH 4.0–4.2 resulted in complete hatching failure, indicating a critical threshold below which development is severely disrupted [64]. It must be emphasized, however, that these pH values (4.0–5.0) are not representative of marine OA, which entails a modest decline from ~8.1 to ~7.8–7.9 in open oceans. Accordingly, these extreme-exposure data should be interpreted as illustrating the upper bounds of pH sensitivity and as a heuristic reference for underlying mechanisms, rather than as predictive evidence of near-future OA impacts.

The developmental success of fish eggs is closely linked to their morphology and quality [64]. Research indicates that exposure to OA-relevant conditions can alter egg size, structural morphology, and membrane integrity, thereby affecting fertilization success and embryonic development. In the Sydney rock oyster Saccostrea glomerata, elevated pCO_2_ significantly reduced fertilization rates and impaired embryogenesis, with effects intensified under combined acidification and warming. Although the precise mechanisms remain to be fully elucidated, these changes have been associated with compromised stability of calcium carbonate structures and activation of cellular stress responses [4,36,56]. Some experimental studies have also shown that high CO_2_ concentrations can affect fish egg development [36,56].

pH fluctuations can alter the normal developmental timeline of eggs. Under acidic conditions, the chorion softens, the egg structure collapses, and elasticity is lost, which hinders normal embryonic development and increases the risk of premature membrane rupture [64]. Chorion degradation further impairs osmotic regulation and pathogen defense, elevating embryonic susceptibility to disease and developmental abnormalities [57,62]—effects that may be exacerbated by thermal stress, as discussed later. Collectively, these observations indicate that pH can influence embryonic development through chorion-mediated pathways. However, given the non-analogous pH ranges, direct extrapolation to marine OA must be made with caution. This underscores the critical need for dedicated studies on early-life stages under environmentally realistic pCO_2_ levels that are directly relevant to projected OA scenarios.

### 3.3. Synergistic Effects: Ocean Acidification Coupled with Temperature Stress

Temperature strongly regulates fish physiology by influencing biochemical processes and reproductive neuroendocrine function [65,66,67]. For example, in salmonids, an increase in water temperature can alter the secretion pattern of GnRH, affect the metabolic clearance of gonadotropins, and change the synthesis of gonadal steroid hormones [68]. Further research has shown that during the critical window of early development, temperature fluctuations can even induce epigenetic reversal of gonadal sex determination [69,70,71]. This phenomenon, known as temperature-dependent sex determination (TSD), demonstrates the exquisite sensitivity of fish developmental processes to environmental cues.

Therefore, the hydrochemical changes caused by OA are likely to have combined or interactive effects with temperature stress, jointly exacerbating the adverse impacts on fish reproduction and early development [21,72]. When fish are exposed to OA and temperature increase concurrently, the combined effects are often more severe than those of individual stressors. While temperature rise can independently alter embryonic development and metabolic rates, acidification often intensifies this thermal pressure, leading to a “double-edged sword” effect where energetic demands exceed available resources [25]. Specifically, these multi-stressor impacts manifest in increased individual activity, higher metabolic consumption, delayed or compromised developmental cycles, and weakening of larvae’s predator-avoidance capabilities [73,74].

It should be noted, however, that the nature of combined responses to warming and acidification is not universally synergistic. Depending on species, physiological endpoint, exposure duration, and acclimation history, interactions can be synergistic, additive, or even antagonistic [25,72]. A formal demonstration of synergy requires a statistical comparison against an expected additive model, and such evidence remains limited across the current literature. Thus, we use the terms “combined” and “interactive” here to describe the overall multi-stressor effects, without implying a universal synergistic relationship.

### 3.4. Early Embryonic Development and Survival Rates

The early embryonic stage of teleost fish is highly vulnerable to environmental fluctuation due to the lack of fully developed homeostatic mechanisms. Research has shown that exposure to acidic conditions can lead to a series of negative effects on fish embryos, such as delayed development, increased occurrence of morphological abnormalities, and significantly elevated mortality rates [14]. The survival rate of larvae and juvenile fish also decreases under elevated pCO_2_ levels, with different species demonstrating significant differences in sensitivity; for instance, some tropical reef fishes exhibit higher tolerance than certain temperate species, revealing a potential species-specific vulnerability pattern under OA stress [12,75].

At the molecular and physiological levels, CO_2_ stress can upregulate the expression of genes related to protein synthesis, ion regulation, and energy production, reflecting an overall increase in their metabolic levels [76]. This metabolic hyperactivity may trigger a critical redistribution of energy within the organism. Acid–base homeostasis consumes significant energy. Embryos therefore distribute resources accordingly from growth and reproduction to maintain pH balance [25]. Such excessive energy consumption to sustain basic functions can bring substantial costs to aquaculture settings and wild population recruitment, ultimately leading to a chain of negative impacts on the reproductive behavior of fish, including reduced fertilization success and delayed or failed egg laying [21,22].

## 4. Research Gaps and Future Directions

### 4.1. Knowledge Gaps and Limitations

In conclusion, while OA remains a primary concern, wild populations are undoubtedly exposed to a mosaic of interacting climate stressors. Yet the current evidence base rests on several interconnected frailties. Most experimental work has been confined to short-term laboratory exposures, which cannot capture the cumulative energetic costs, transgenerational plasticity, or life-history adjustments that manifest over years and multiple generations in nature [8,77]. Mechanistically, the proposed pathways—GABA_a_ disruption [37,78], olfactory impairment [53,79,80], and circadian interference [27,35]—have each been studied under static conditions, leaving their relative dominance and potential synergies in fluctuating environments essentially unconstrained. Moreover, the reproductive impacts of climate change extend well beyond acidification itself: altered temperature and rainfall regimes already shift spawning phenology in temperate [20], tropical [81,82], and reef-associated species [83], while poleward migrations expose fish to novel photoperiods that may truncate breeding seasons [73,84]. Even the direction of OA’s effect on reproductive output is debated—some studies report enhanced female fecundity [85], whereas others point to impaired male mating behavior [55]—and the ecological consequences of these changes remain unknown. Superimposed on these are salinity fluctuations [27,86] and oxygen depletion [25,74], both of which directly compromise gonadal development and gamete quality, yet their combined effects with warming and acidification are rarely investigated [25,74,87].

Moving forward, research must pivot toward integrated, ecologically realistic frameworks. Long-term, multigenerational field monitoring—ideally paired with mesocosm experiments that simulate natural diel and seasonal pCO_2_ variability—is urgently needed to validate laboratory findings and document real-world fitness outcomes [8,77,81]. Mechanistic studies should pinpoint the molecular intersections between acid–base regulation and reproductive neuroendocrinology, clarifying whether observed behavioral changes are reversible or heritable [27,35,37,53,78,79,80]. Critically, experimental designs must systematically incorporate factorial combinations of warming, deoxygenation, salinity change, and acidification, because their joint effects on reproductive success are virtually uncharted [25,74,86,87]. Predictive models, which currently rely heavily on coarse emission scenarios [7,8], must be refined to include interaction coefficients and to extend beyond marine environments; freshwater and brackish systems face additional acidification from acid rain and runoff, as well as more erratic flow regimes, yet they remain severely under-modelled [88,89,90]. Finally, the documented sensitivity of spawning to temperature, rainfall, lunar cycles, and photoperiod [20,73,81,82,83,84] underscores that phenological shifts must be embedded in any future assessment of reproductive success. Only by embracing this multifaceted, mechanism-informed, and habitat-inclusive agenda can we generate the robust understanding required to anticipate and mitigate the compounding threats to fish reproduction under accelerating climate change.

### 4.2. Importance of Long-Term and Multi-Generational Studies

Long-term and multigenerational studies are crucial for understanding the full extent of OA impacts on fish reproduction. Such research can reveal the potential for selective adaptation to severe acidification conditions and help forecast how wild fish populations will respond to future environmental changes [13,35]. The adaptive potential of a given population depends on its genetic variability, and research indicates that OA exerts varying degrees of behavioral impact on different individuals within a single species. Importantly, these individual differences can be transmitted across generations [35], as illustrated by the case of the spiny chromis (*Acanthochromis polyacanthus)* detailed below.

Evidence suggests that some fish species possess the inherent genetic capacity to adapt to OA. A foundational study on the spiny damselfish (*Acanthochromis polyacanthus*) classified parents as either CO_2_-sensitive or CO_2_-tolerant based on olfactory behavior. Genomic, transcriptomic, and proteomic analyses of their offspring revealed that progeny from tolerant parents exhibited distinct molecular profiles in brain tissue after high-CO_2_ exposure [35]. These transgenerational molecular features indicate that pre-existing variation in CO_2_ sensitivity within a population can provide the raw material for natural selection, underscoring the potential for evolutionary rescue in a changing ocean [9,35,73].

While transgenerational studies offer hope, other research highlights a more immediate threat OA poses to fish reproduction. A key concern is the direct impact of altered pH on gamete function. Sperm motility, essential for successful fertilization, is highly sensitive to the chemical environment [52,58]. Studies in rainbow trout have established a clear causal relationship between decreased seminal pH and loss of sperm motility. This physiological mechanism is highly relevant to OA scenarios, where the projected decline in ocean pH could directly interfere with sperm physiology in external fertilizers. By altering the chemical cues and conditions required for sperm activation and movement, OA has the potential to severely impact fertilization success and subsequent recruitment, independent of a population’s long-term adaptive potential [52]. Low pH (7.5) alone does not decrease sperm vitality for hours, but the addition of lactic acid causes a significant rapid, reversible motility decline. This effect was only observed at lactic acid concentrations not exceeding 10 mM. Thus, the combined effects of pH drop and lactate accumulation in the gonads post-mortem are likely responsible for the irreversible loss of rainbow trout sperm motility over time [52].

### 4.3. Population-Level and Ecosystem Consequences of Reproductive Impairment Under Ocean Acidification

OA impacts on individual fish reproduction have far-reaching consequences that extend beyond the physiological and behavioral levels to affect population dynamics, community structure, and entire ecosystem functioning [23,26]. The reproductive dysfunction induced by OA in fish species can cascade through marine food webs, ultimately threatening global fishery productivity and marine ecosystem services essential for human food security and biodiversity conservation. Understanding these population-level and ecosystem-wide impacts is critical for developing comprehensive management strategies and conservation policies [9,23]. A central concern regarding OA’s impact on fish reproduction is its potential to reduce population recruitment rates, which are fundamentally dependent on successful reproduction and larval survival. In many fish species, recruitment is a critical population bottleneck; even small reductions in spawning success, fertilization rates, or early-stage survival can translate into substantial population declines over multiple generations. When elevated pCO_2_ suppresses sex hormone synthesis, impairs gonadal maturation, or reduces gamete quality, this results in the number of viable offspring produced per spawning event decreasing significantly. For species with limited reproductive opportunities (e.g., those with restricted spawning seasons or long generation times), these reproductive failures can accumulate rapidly, leading to population crashes [16].

Empirical evidence from field observations and laboratory experiments reveals that OA-induced recruitment failure is particularly severe in species with specific reproductive strategies. For instance, fish that release gametes into the water column rather than providing parental care are especially vulnerable because their reproductive success depends critically on precise sperm–egg encounter rates and successful fertilization in the water column. When sperm motility is compromised by reduced pH and egg quality is diminished by acidification stress, fertilization success plummets even if spawning behavior itself remains unaffected. This creates a hidden reproductive bottleneck that may not be immediately apparent through behavioral observations alone [12,23].

Furthermore, the transgenerational effects of parental exposure to OA can compound recruitment problems. Offspring from parents stressed by acidification conditions often exhibit reduced size at hatching, delayed development, increased susceptibility to predation, and impaired antipredator behaviors. These factors collectively reduce larval survival chances. In coral reef fishes, such as damselfish and clownfish, these combined effects have been shown to reduce recruitment by 50–80% under simulated future OA scenarios, with consequences that persist for multiple subsequent generations even when fish are removed from acidified conditions [35].

OA does not affect all fish species equally; rather, it creates differential selective pressures that favor some species over others based on their physiological tolerance and reproductive strategies. Species with robust acid–base regulation capacity, large energy reserves, and flexible reproductive timing may tolerate OA stress more effectively than species with narrow physiological tolerance ranges. This heterogeneous response generates conditions for major shifts in species composition within communities, a process termed community restructuring [9].

In marine ecosystems characterized by high endemism (i.e., coral reef environments), these shifts can be particularly dramatic. Species that currently dominate in abundance and biomass may decline substantially, whereas previously rare or minor taxa might expand their populations. The net result is fundamentally altered community composition, with uncertain consequences for ecosystem stability and function. For example, in some coral reef communities, more aggressive and competitive fish species appear to tolerate OA conditions better than territorial herbivorous species, potentially shifting the balance of interspecific competition and altering the structure of fish assemblages [26].

These community-level changes are particularly concerning because they frequently involve the losses of specialist species alongside the expansion of generalist taxa, a pattern that generally reduces biodiversity and ecosystem stability [26]. Furthermore, if the species most severely impacted by OA include those occupying key ecological roles (e.g., apex predators, planktivores, or herbivores), ecosystem-wide trophic disruptions can cascade through food webs, affecting organisms far removed from the initial stressor [91].

Beyond direct effects on reproduction, OA-induced changes in fish behavior and sensory perception profoundly disrupt predator–prey interactions, with significant implications for food web structure and stability [9]. The impairment of olfactory abilities and the disruption of predator avoidance behaviors in larvae and juveniles exposed to elevated pCO_2_ levels increase their vulnerability to predation, effectively shifting predator–prey encounter rates and predation pressure dynamics [16,53]. Enhanced predation on OA-susceptible species can amplify population declines, particularly for species whose larvae and juveniles already suffer from reduced growth and development rates [12].

Large predatory species, which have longer generation times and greater reproductive demands, may decline relative to smaller prey if disproportionately affected by OA. This would alter trophic structure, reducing top predators and potentially triggering trophic level restructuring, where mid-trophic species face less predation but more competition and resource depletion [26]. The net ecosystem consequences are difficult to predict precisely but likely include reduced energy transfer efficiency through food webs, decreased overall ecosystem productivity, and potentially boom–bust population cycles as ecosystems attempt to reorganize around new energy and nutrient dynamics [26].

The cumulative effects of OA on fish reproduction, population dynamics, and community structure translate directly into reduced fishery productivity, with severe socioeconomic consequences for human populations dependent on marine resources [23]. Global fish stocks are already stressed by overfishing, habitat degradation, and climate change-driven warming; OA represents an additional stressor that synergizes with these existing pressures to reduce the yield potential of wild fish populations [16].

Economically important species like groupers, snappers, tuna, and salmon are also being affected by OA. The reproductive impairment by OA of economic species can lead to earlier recruitment failure, lower peak abundance, and more volatile population fluctuations. Small-scale fishing communities and developing nations that depend heavily on subsistence and artisanal fisheries are particularly vulnerable to such declines, as they lack the economic resources to diversify food sources or adapt to changing fish availability [23]. In regions such as Southeast Asia and the Pacific Islands, where fish provide over 50% of dietary protein, OA-driven reductions in fish production threaten nutritional security and livelihoods for millions of people [23,92].

Increased variability and unpredictability of fish populations under combined OA and warming stress create management challenges for fishery regulators [23]. Traditional fishery management approaches based on historical population dynamics and stable recruitment patterns become unreliable when underlying biological processes are fundamentally altered. This necessitates the development of novel adaptive management frameworks that can accommodate rapid environmental change and highly variable population responses [91].

The resistance and resilience of marine ecosystems to OA-induced reproductive disruption depend critically on the diversity of species present, the strength of key ecological interactions, and the capacity of organisms to acclimate or adapt [91]. Ecosystems with high biodiversity and functional redundancy are generally more resistant to species losses and better able to maintain ecosystem functions despite perturbations. This resistance is only possible when multiple species occupy similar ecological roles. Conversely, ecosystems with low diversity or ecosystems dominated by a few key species are more vulnerable to reproductive failure in those species, as there are fewer alternative species to compensate functionally [91].

The accelerating pace of OA (pH declining 0.4 units by 2100 under high-emission scenarios) may challenge the adaptive capacity of many fish species, particularly those with long generation times and limited plasticity in reproductive timing. Under such rapid environmental change, the probability of maladaptation increases substantially. Fish populations may be unable to evolve reproductive strategies aligned with new environmental conditions faster than their habitat shifts, creating a “moving target” problem where adaptation always lags behind environmental change [77].

OA rarely occurs in individual state; it typically co-occurs with ocean warming, deoxygenation, and alterations in nutrient availability [8]. These synergistically affect reproductive processes and ecosystem functioning [72]. The simultaneous exposure to multiple stressors reduces survival margins for populations already compromised by single-stressor effects and can trigger unexpected, non-additive (synergistic) impacts that are difficult to predict from single-stressor experiments [72]. For instance, the combination of moderate OA and warming can trigger much greater reproductive dysfunction than either stressor alone, a phenomenon observed in multiple fish species and attributed to the interactive effects of multiple stressors on energy metabolism and neuroendocrine function [21,22,74].

The population-level and ecosystem-wide consequences of OA-induced reproductive impairment underscore the urgent need for integrated, multi-scale conservation strategies [9]. Individual mitigation strategies should focus on reducing anthropogenic stressors. These include fishing pressure, habitat degradation, and pollution. These may give chance to partially offset the negative impacts of OA. By reducing extraneous mortality and habitat loss, populations may retain sufficient reproductive capacity to maintain viability despite OA-induced reductions in recruitment [9].

At the ecosystem level, the establishment of well-managed marine protected areas (MPAs) that preserve ecosystem structure and maintain populations of key species is critical [91]. Such reserves can serve as protection for sensitive species, maintain ecosystem connectivity, and preserve evolutionary potential for long-term adaptation. However, traditional fixed-boundary MPAs may be insufficient under rapid climate change; dynamic MPA networks that shift boundaries in response to species range movements and changing environmental conditions are likely necessary for long-term conservation efficacy [91].

Ultimately addressing OA and its consequences requires a dramatic and urgent reduction in anthropogenic carbon dioxide emissions [23]. While local-scale adaptation and mitigation strategies are valuable and necessary components of climate response, they cannot substitute for global reduction in greenhouse gas emissions. Achieving the Paris Agreement’s target of limiting global warming to 1.5–2 °C above pre-industrial levels could substantially reduce the rate and magnitude of OA, affording marine organisms and ecosystems a greater opportunity to acclimate and adapt to shifting environmental changes [23].

### 4.4. Strategies for Adaptation and Mitigation

Safeguarding marine biodiversity in the face of OA hinges on two key priorities: understanding the adaptive potential of fish populations and developing effective mitigation strategies. Immediate conservation efforts should prioritize habitat restoration to bolster ecosystem resilience, coupled with global measures to reduce CO_2_ emissions and the creation of marine protected areas (MPAs) to mitigate other environmental pressures [9,23]. While selective breeding of OA-resistant strains may hold promise for aquaculture, its direct application to wild marine populations is not straightforward and carries ecological and genetic risks, such as loss of genetic diversity and potential introgression into wild stocks; thus, it should be considered cautiously and primarily within controlled rearing contexts. The physiological response of fish to OA and warming is highly variable, shaped by species-specific traits like acid–base regulation capacity, calcification needs, and reproductive investment strategies, which also change throughout their life cycle. However, the current research landscape is dominated by short-term, single-generation studies examining isolated life stages [25,27]. This approach leaves a critical gap in our knowledge of long-term adaptation and evolutionary processes. A paradigm shift is therefore needed, leveraging quantitative genetics and genomic tools to comprehensively assess the adaptive capacity of fish to ongoing environmental change [13,35].

## 5. Conclusions

Ocean acidification is a multi-dimensional threat to marine fish reproduction. This complex threat influences processes from gametogenesis to early embryonic development through coordinated physiological, endocrine and molecular effects. Our review identifies three principal mechanisms underlying these disruptions. First, OA affects the hypothalamic–pituitary–gonadal (HPG) axis, leading to reduced synthesis of key reproduction steroid hormones, especially 17β-estradiol, which in turn suppresses vitellogenesis and delays or prevents gonadal maturation. Second, it alters gamete function by reducing sperm motility, which is optimal within a pH range of 8.0 to 10.0, and by compromising egg quality. These disruptions are influenced by altered calcium signaling, effects on membrane quality, and acid-base regulation. Third, these compromises are further intensified under coupling stressors such as elevated temperature, where combined stress conditions produce more prominent reproductive dysfunction than ocean acidification alone.

Although some species may exhibit adjustment due to genetic variation in CO_2_ responses, the projected rate of ocean acidification, approximately a 0.4 unit decline in pH by 2100 under the high-emission scenario RCP 8.5, is likely to exceed the plasticity capacity of many fish populations, particularly those with narrow physiological tolerances These damage in reproduction may therefore reach to population-level consequences, which may include recruitment, shifts in community structure, altered predator-prey interactions, and declining fisheries productivity. Overall, these effects pose serious risks to marine biodiversity, ecosystem stability, and global food security. Addressing these challenges requires urgent and integrated conservation strategies, including control of carbon emissions, and the addition of marine protected areas.

## Figures and Tables

**Table 1:** Summary of key findings, practical implications, and research gaps in ocean acidification effects on fish reproduction.

Aspect	Key Findings	Practical Implications	Remaining Knowledge Gaps
HPG Axis Disruption	OA suppresses *igf-1* and *crh* system gene expression; impairs GnRH and gonadotropin release via GABA_A_ receptor-mediated inhibition; reduces 17β-estradiol synthesis and vitellogenesis.	Reduced reproductive output in aquaculture and wild fisheries; potential for delayed maturation in commercially important species.	Molecular mechanisms linking acid–base compensation to HPG axis disruption remain unclear; no multi-generational studies on HPG axis disruption to population-level recruitment failure.
Gonadal Development	pH reduction inhibits P450 aromatase (CYP19A1A) expression through *dax1* gene, impairing androgen-to-estrogen conversion; energy trade-off hypothesis reduces energy allocation to reproduction.	Potential for skewed sex ratios in wild populations; reduced fecundity in female fish.	Whether *dax1*-mediated suppression is conserved across taxa; long-term effects on population sex ratios.
Gamete Quality & Function	Sperm motility optimal at pH 8.0–10.0; motility reduced at pH < 7.4; ovarian fluid pH critical for egg survival (≥80% survival at pH 8.44–8.57); Ca^2+^ signaling disruption affects egg activation.	Shortened fertilization windows for broadcast spawners; reduced hatchery success; need for pH monitoring in aquaculture facilities.	Species-specific pH optima for gamete function; mechanisms of Ca^2+^ signaling disruption; effects on egg quality beyond survival.
Embryonic Development	Acidic conditions cause chorion softening, delayed hatching (up to 6 days in Atlantic salmon), morphological abnormalities, and elevated mortality; embryos lack fully developed homeostatic mechanisms.	Increased larval mortality in wild populations; reduced recruitment success; implications for hatchery protocols.	Interactive effects with other stressors during critical developmental windows; epigenetic effects across generations.
Multi-Stressor Synergies	OA + warming produce greater reproductive dysfunction than either stressor alone; temperature can induce epigenetic sex reversal (TSD); combined stressors increase metabolic demands and reduce energy for reproduction.	Climate change projections underestimating impacts if ignoring synergies; need for integrated management approaches.	Non-additive effects of OA + warming + deoxygenation + salinity changes; thresholds for synergistic effects across species.
Transgenerational Adaptation	Some species show heritable CO_2_ tolerance (e.g., *Acanthochromis polyacanthus*); genetic variation exists within populations for OA sensitivity.	Potential for selective breeding of tolerant strains; hope for adaptation in some populations.	Extent of adaptive capacity across species; trade-offs associated with CO_2_ tolerance; rate of adaptation vs. rate of environmental change.
Population and Ecosystem Consequences	Reproductive impairment may lead to recruitment failure, lower peak abundance, volatile population fluctuations; impacts on food webs and predator–prey interactions.	Threats to food security, especially in developing nations where fish provide >50% dietary protein; challenges for fisheries management; need for dynamic MPAs.	Population-level consequences of reproductive disruption not yet quantified; ecosystem modeling under multi-stressor scenarios.
Geographic and Taxonomic Variation	Tropical reef fishes show higher tolerance than some temperate species; species-specific responses to OA; seasonal breeding species particularly vulnerable.	Regional vulnerability assessments needed; differential management strategies for tropical vs. temperate fisheries.	Mechanisms underlying species-specific tolerance; impacts on poleward-shifting species experiencing novel photoperiods.
Behavioral Disruption	Increased aggression and disrupted courtship in damselfish; olfactory disruption affects chemical cue detection; potential impacts on mate choice and spawning synchrony.	Reduced mating success; altered social structures; implications for stock assessments relying on behavioral cues.	Neurobiological mechanisms linking OA to behavioral changes; ecological significance of behavioral disruptions.
Conservation and Management	MPAs may provide refugia; reducing local stressors (fishing pressure, habitat degradation, pollution) can offset some OA impacts; global CO_2_ reduction is essential.	Need for adaptive management frameworks; dynamic MPA networks; integration of OA into fisheries stock assessments.	Effectiveness of MPAs under rapid climate change; cost–benefit analyses of adaptation strategies.

**Table 2:** Summary of species-specific reproductive responses to ocean acidification conditions.

Species	Ph/pCO_2_ Level	Exposure Duration	Reproductive Endpoint	Effect Size/Key Finding	Reference
*Cyprinus carpio var. color* (multiple spp.)	Near-future CO_2_ (~850 µatm)	Chronic	Reproductive output, CRH expression	Elevated CRH correlated with reduced output	Miller et al. 2015 [21]
Three-spined stickleback (*Gasterosteus aculeatus*)	Elevated pCO_2_ (~1000 µatm)	Multi-year (long-term)	Gonadal development, sexual maturation	Significantly reduced, delayed maturation	Devergne et al. 2023 [22]
Zebrafish (*Danio rerio*)	pH 7.5 (control: 8.1)	Juvenile stage	IGF-1 mRNA expression	Suppressed expression, impaired growth	Feng et al. 2024 [29]
Spiny damselfish (*Acanthochromis polyacanthus*)	Elevated pCO_2_	Transgenerational	Olfactory behavior, molecular profiles	Distinct molecular signatures in tolerant offspring	Schunter et al. 2016 [35]
Spiny damselfish (*Acanthochromis polyacanthus*)	Elevated pCO_2_	Transgenerational	Olfactory behavior, molecular profiles	Distinct molecular signatures in tolerant offspring	Schunter et al. 2016 [35]
River trout/Brown trout	pH 5.5	In vitro activation	Sperm motility	Maintained 31–38% motility at acidic pH	Brzyszcz et al. 2024 [52]; Ciereszko et al. 2010 [56]
European eel (*Anguilla anguilla*)	pH > 7.8 (hatchery control)	Embryonic development	Survival and development	Significantly improved at pH > 7.8	Sganga et al. 2022 [57]
Atlantic salmon (*Salmo salar*)	pH 4.5–5.0	Embryonic development	Hatching time, hatching success	Delayed hatching by up to 6 days; failed at pH 4.0–4.2	(Cited in Section 3.2)

## Data Availability

No new data were created or analyzed in this study. Data sharing is not applicable to this article.

## Glossary

HPG axis (Hypothalamic–Pituitary–Gonadal axis): The central neuroendocrine regulatory system controlling reproduction in vertebrates. In fish, the hypothalamus secretes gonadotropin-releasing hormone (GnRH), which stimulates the pituitary gland to release gonadotropins (FSH and LH). These hormones then act on the gonads to regulate gametogenesis and steroid hormone synthesis. Vitellogenesis: The process of yolk accumulation in developing oocytes (egg cells). This energy-intensive process provides essential nutrients for embryonic development and is tightly regulated by estrogen and other endocrine signals. Suppression of vitellogenesis under stress conditions directly reduces offspring survival potential. Hypercapnia: A condition of abnormally elevated carbon dioxide (CO_2_) levels in the blood or tissues. In the context of ocean acidification, fish exposed to high environmental pCO_2_ experience internal hypercapnia, which triggers compensatory acid-base regulatory mechanisms that consume metabolic energy. P450 aromatase (P450arom, CYP19a1a): A key enzyme encoded by the cyp19a1a gene that catalyzes the conversion of androgens (e.g., testosterone) to estrogens (e.g., estradiol). This enzyme is essential for female sex determination, ovarian differentiation, and the maintenance of reproductive function in both sexes. Dax1 (Dosage-sensitive sex reversal, Adrenal hypoplasia congenita, X-linked gene 1): An orphan nuclear receptor that functions as a transcriptional repressor. In the reproductive context, Dax1 negatively regulates the expression of P450 aromatase, thereby controlling estrogen synthesis. Alterations in Dax1 activity under acidic conditions may disrupt normal gonadal development. GABA_A_ receptor (Gamma-aminobutyric acid type A receptor): A ligand-gated ion channel receptor in the central nervous system that mediates inhibitory neurotransmission. In fish, the GABA_A_ receptor is implicated in the regulation of GnRH release. Ocean acidification-induced disruption of intracellular chloride homeostasis may impair GABA_A_ receptor function, thereby suppressing reproductive signaling. Otolith: Calcified structures located in the inner ear of fish that function in balance, orientation, and sound detection. Because otolith formation depends on calcium carbonate deposition, ocean acidification can alter otolith size, shape, and symmetry, potentially impairing larval swimming behavior, predator avoidance, and spatial navigation.
